# Ginsenoside Rg3-induced EGFR/MAPK pathway deactivation inhibits melanoma cell proliferation by decreasing FUT4/LeY expression

**DOI:** 10.3892/ijo.2015.2886

**Published:** 2015-02-10

**Authors:** Xiu Shan, Faisal Aziz, Li Li Tian, Xiao Qi Wang, Qiu Yan, Ji Wei Liu

**Affiliations:** 1 Department of Oncology, The First Affiliated Hospital of Dalian Medical University, Dalian 116011, P.R. China; 2 Department of Biochemistry and Molecular Biology, Liaoning Provincial Core Laboratory of Glycobiology and Glycoengineering, Dalian Medical University, Dalian 116044, Liaoning, P.R. China; 3 Department of Dermatology, Northwestern University, Feinberg School of Medicine, Chicago, IL 60611, USA

**Keywords:** cell proliferation, fucosyltransferase IV, Lewis Y, melanoma, Rg3

## Abstract

Malignant melanoma is a destructive and lethal form of skin cancer with poor prognosis. An effective treatment for melanoma is greatly needed. Ginsenoside Rg3 is a herbal medicine with high antitumor activity. It is reported that abnormal glycosylation is correlated with the tumor cell growth. However, the antitumor effect of Rg3 on melanoma and its mechanism on regulating glycosylation are unknown. We found that Rg3 did not only inhibit A375 melanoma cell proliferation in a dose-dependent manner, but also decreased the expression of fucosyltransferase IV (FUT4) and its synthetic product Lewis Y (LeY), a tumor-associated carbohydrate antigen (TACA). Knocking down FUT4 expression by siRNA dramatically reduced FUT4/LeY level and inhibited cell proliferation through preventing the activation of EGFR/MAPK pathway. Consistently, the inhibitory effect of the Rg3 and FUT4 knockdown on melanoma growth was also seen in a xenograft melanoma mouse model. In conclusion, Rg3 effectively inhibited melanoma cell growth by downregulating FUT4 both *in vitro* and *in vivo*. Targeting FUT4/LeY mediated fucosylation by Rg3 inhibited the activation of EGFR/MAPK pathway and prevented melanoma growth. Results from this study suggest Rg3 is a potential novel therapy agent for melanoma treatment.

## Introduction

Malignant melanoma is the leading cause of skin cancer-related death (80%) worldwide, mainly due to its high proliferation capability, metastatic properties and lack of effective treatment (1). According to the World Health Organization (WHO), the incidence of melanoma is rising faster than that of any other type of cancer worldwide (2). There are a limited number of efficacious therapies that are current available, including interferon α-2b, interleukin (IL)-2, the anti-CTLA-4 antibody, ipilimumab, the BRAF inhibitors, vemurafenib and dabrafenib, as well as a MEK inhibitor, trametinib. In addition, either significant immune related adverse effects or short-lived durable responses of these available treatments further limited their therapeutic application (3–5). Therefore, there is still a need to find and identify potential low toxicity and sensitive drugs, which recognize specific molecular target to strengthen the efficacy in the treatment of melanoma.

Lewis Y (LeY) is a difucosylated oligosaccharide with the chemical structure [Fucα1→2Galβ1→4(Fucα1→3)GlcNAcβ1→R], carried by glycoconjugates (glycoproteins and glycolipids) on the cell surface. LeY is a tumor-associated carbohydrate antigen (TACA) and its abnormal expression is frequently found in various cancers, such as breast, pancreas, gastric, ovarian and skin cancers (6–9). Several reports suggested that abnormal expression of LeY was associated with tumor growth, metastasis, angiogenesis and drug resistance (10–13). Monoclonal antibody 692/29 targeting LeY showed good antitumor responses (14). The activation of epidermal growth factor receptor (EGFR) in skin cancers is closely related to the carcinogenic events including cell proliferation, migration and invasion (15). LeY oligosaccharide antigen linked to EGFR plays a critical role in dimerzation and activation of EGFR as well as downstream of the EGFR/MAPK signaling pathway. Therefore, the inhibition of LeY synthesis and LeY mediated activation of EGFR/MAPK signaling pathway play an important role in the treatment of cancer.

The synthesis of tumor-associated carbohydrate antigens, including LeY, is controlled by the specific glycosyltransferases. Fucosyltransferases (FUTs) are the key enzymes catalyzing the synthesis of fucosylated glycans. FUTs gene family contains 1, 2-, 1, 3/4-, and 1, 6-linkages, which catalyze the transfer of L-fucose from GDP-fucose to their acceptors. At least eight 1, 3/4-FUT genes have been identified: FUT3, 4, 5, 6, 7, 9, 10 and FUT11 (16,17). The 1, 3-fucosylation of LeY is catalyzed by fucosyltransferase IV (FUT4). Increased FUT4 expression has been reported in many cancers, such as gastric, colorectal, and lung cancer (18–20). Our previous study showed that high proliferative ability skin cancer A431 cells carried a higher level of FUT4 than low proliferative capability SCC12 cells (21). Moreover, high expression of FUT4 promoted cell proliferation by augmenting the synthesis of LeY (22), while suppressing the expression of FUT4 by RNAi technology reduced the synthesis of LeY and inhibited cell growth (9). Therefore, it is critical to find effective drugs which can decrease the synthesis of FUT4/LeY and inhibit tumor growth.

Ginsenoside Rg3 is the main active component in ginseng. Rg3 has a wide range of pharmacological and therapeutic effects, including anti-inflammation, anti-fatigue, immune stimulation and anticancer. Previous reports showed that Rg3 had anticancer effect in gastric, breast, colon and hepatocellular cancers (23–26). Currently, Rg3 is used clinically to treat late-stage non-small cell lung cancer (NSCLC) in China. The underlying anticancer mechanisms of Rg3 contain anti-proliferation, induce apoptosis, anti-metastasis and anti-angiogenesis (26–28). However, the mechanism of Rg3 on melanoma cell proliferation, and its potential role in regulation of FUT4 and LeY expression on cell proliferation have not been reported.

In the present study, we found that Rg3 significantly inhibited melanoma cell growth through inhibiting EGFR phosphorylation with FUT4/LeY low expression *in vitro* and *in vivo*. We suggest that Rg3 deactivation of the EGFR/MAPK pathway through downregulating FUT4/LeY expression performs a key role in the treatment of melanoma.

## Materials and methods

### Ethics statement

All animal work performed in this study was approved by the Animal Ethics Committee of Dalian Medical University. Moreover, the detail protocols and experimental processes conformed to the Experimental Animal Management Regulations of Dalian Medical University.

### Reagents and antibodies

The 20 (R)-Rg3 was provided by Dalian Fu Sheng Pharmaceutical Co. (Dalian, China). A solution of Rg3 as freshly prepared in DMEM (500 μg/ml) and filtered by 0.22-μm membranes. It was diluted with cell culture media to final concentration in different treatments. DMEM, fetal bovine serum (FBS), TRIzol and Lipofectamine™ 2000 reagents were purchased from Invitrogen (Camarillo, CA, USA). DMSO and MTT were purchased from Sigma-Aldrich (St. Louis, MO, USA). The enhanced chemiluminescence (ECL) assay kit was purchased from Amersham (Pittsburgh, PA, USA). Anti-rabbit FUT4, PCNA, p-ERK1/2, ERK, β-actin, HRP-conjugated anti-mouse IgM, HRP-conjugated anti-rabbit and anti-mouse IgG antibodies were purchased from Proteintech group (Wuhan, China). EGFR and p-EGFR were purchased from Cell Signaling Technology (Boston, MA, USA). FITC conjugated goat anti-mouse IgG and TRITC conjugated goat anti-rabbit IgG were purchased from Santa Cruz Biotechnology (Santa Cruz, CA, USA). HRP-Ulex Europaeus (UEA) lectin was purchased by EY Laboratories (San Mateo, CA, USA), which preferentially recognizes the total fucose. Mouse anti-Giantin Golgi marker antibody and mouse anti-LeY antibody (BG-8) were purchased from Abcam (Cambridge, UK). AG1478 inhibitor was obtained from Sigma (St. Louis, MO, USA). Coomassie protein assay reagent was purchased from Bio-Rad (Hercules, CA, USA).

### Cell culture

Human melanoma cell line A375 (original commercial source from American Type Cell Culture, ATCC, Manassas, VA, USA) was a kind gift from Dr Xiao-Qi Wang (Department of Dermatology, Northwestern University, IL, USA) and cultured in DMEM with 10% FBS, 100 U/ml penicillin and 100 μg/ml streptomycin; maintained at 37°C under 5% CO_2_ in humidified air.

### Transient transfection

A375 cells (1×10^5^) were trypsinized and seeded onto 6-well plates. When cells reached 70–80% confluence, FUT4 siRNA was transiently transfected into A375 cells using Lipofectamine™ 2000 Reagent following the manufacturer’s instructions (Invitrogen, Carlsbad, CA, USA). The transfection reagent was removed after 5 h and the cells were harvested after 48 h.

### Cell viability assay

MTT assay was performed to detect the effect of Rg3 on A375 melanoma cell proliferation. In brief, cells (2×10^3^/well) were plated in 96-well plates. Twenty-four hours later, cells were treated with different concentrations of Rg3 (0, 25, 50 and 100 μg/ml) for another 24 h before MTT was added into the culture medium (0.5 mg/ml). After incubation for 4 h at 37°C, the cells were lysed with DMSO at room temperature for 10 min. The absorbance was measured at 490 nm on a microplate reader (Bio-Rad). The test was repeated three times.

### Colony forming assay

Cells (1×10^3^ cells/well) were plated in 6-well plates containing DMEM with 10% FBS at 37°C. After 24 h, cells were treated with different concentrations of Rg3 (0, 25, 50 and 100 μg/ml) for 24 h and then cells were allowed to grow for 7 days in the absence of Rg3. Cells were fixed and stained with crystal violet (0.5%) for 20 min at room temperature. Images were captured with the inverted microscope (Olympus IX71, Japan).

### Immunoblotting

Cells were washed with PBS (pH 7.4), and incubated with lysis buffer [1% Triton X-100, 150 mM NaCl, 10 mM Tris, pH 7.4, 1 mM ethylenediaminetetraacetic acid (EDTA), 1 mM ethyleneglycol tetraacetic acid (EGTA), pH 8.0, 0.2 mM Na_3_VO_4_, 0.2 mM phenylmethylsulfonyl fluoride, 0.5% Nonidet P-40] on ice for 15 min. The cell lysates were clarified by centrifugation at 9000 × g for 10 min and the supernatants were collected. Protein concentration was determined with the Coomassie protein assay reagent (Bio-Rad) using BSA as a standard. Cell lysates (50–100 μg/ml) were separated by an 8–12% SDS-PAGE gel electrophoresis. Samples were transferred electrophoretically to nitrocellulose membranes or PVDF (0.2 μm), blocked with TTBS (50 mM Tris-HCl, pH 7.5, 0.15 M NaCl, 0.1% Tween-20) containing 5% fat-free dry milk followed by overnight incubation at 4°C with anti-FUT4 (1:1,000), anti-LeY (1:200), anti-PCNA (1:1,000), anti-EGFR (1:500), anti-pEGFR (1:500), anti-ERK1/2 (1:500) and anti-pERK1/2 (1:500). The specific antibody binding was detected using HRP-conjugated anti-rabbit or anti-mouse antibody (1:2,000) for 45 min at room temperature. β-actin antibody (1:1,000) was used to confirm the equal loading. Immunoreactive proteins were visualized with ECL detection system and data were analyzed by Image Lab.

### Indirect immunofluorescent staining

Cells were grown on glass coverslips. After washing with PBS, cells were fixed in 4% paraformaldehyde-PBS for 30 min and followed treatment with 0.1% Triton X-100 for 10 min at 4°C. After being blocked with goat serum for 30 min (37°C), cells were incubated with rabbit anti-FUT4 (1:100) and mouse anti-Giantin Golgi marker antibody (1:100) at 4°C overnight. TRITC-conjugated goat anti-rabbit IgG and FITC-conjugated goat anti-mouse IgG secondary antibody were incubated for 1 h at room temperature. Images were captured with the inverted and confocal microscope (Olympus IX71, Japan and LEICA TCS SP5 II, Germany).

### Xenograft tumor mouse model

Male nude mice (Balb/c-nu/nu) were obtained from Animal Center (Dalian Medical University). The animals (4–6 weeks) were maintained under sterile conditions during the entire experimental period. A375 cells (2×10^6^) suspended in 0.2 ml PBS were injected subcutaneously into the right flank. After 7 days of tumor development, mice were randomly divided into 5 different groups (n=6/group). Rg3 20 mg/kg of body weight, FUT4 siRNA plasmid (siFUT4) (6 mg/kg) or Rg3 (20 mg/kg) plus siFUT4 (6 mg/kg) were subcutaneously administered for 3 weeks with time interval of 48 h. The mice treated with vehicle or empty vector only served as controls. Tumor volume was measured by Vernier calipers every other day after tumor inoculation. The tumor volume was calculated according to the formula (volume = 1/2 length × width^2^). At the end of the experiment (the 30th day), the tumor mass was weighed.

### Immunohistochemical staining

The expression of FUT4 was analyzed by immunohistochemistry (IHC) using paraffin-embedded melanoma patient tissues. Serial sections (4 μm each) were prepared, deparaffinized in xylene and rehydrated in a graded alcohol. After microwaved for 20 min in citrate buffer to expose the antigen and washed with PBS, slides were incubated in 3% H_2_O_2_ for 10 min at room temperature to block endogenous peroxidase activity. Non-specific binding was blocked with goat serum at room temperature for 30 min before incubation overnight at 4°C with rabbit IgG FUT4 (1:100). After extensive washing with PBS, sections were incubated with respect secondary antibody for 30 min at room temperature. The signal was visualized with peroxidase-labeled streptavidin complexes DAB and the sections were briefly counterstained with hematoxylin. Yellowish-brown stain indicated a positive result. The negative control was generated by replacing the primary antibody with isotype IgG. Slides were mounted and visualized on an inverted microscope (Nikon Ti-DS, Japan).

### Statistical analysis

Each experiment was repeated 3 times and results presented as the mean ± SEM. P<0.05 was considered to be significant and P<0.01 was considered to be highly significant. Statistical software SPSS ver. 16 was used for analyzing the data.

## Results

### Rg3 inhibits cell growth in A375 melanoma cells

**Figure 1:**
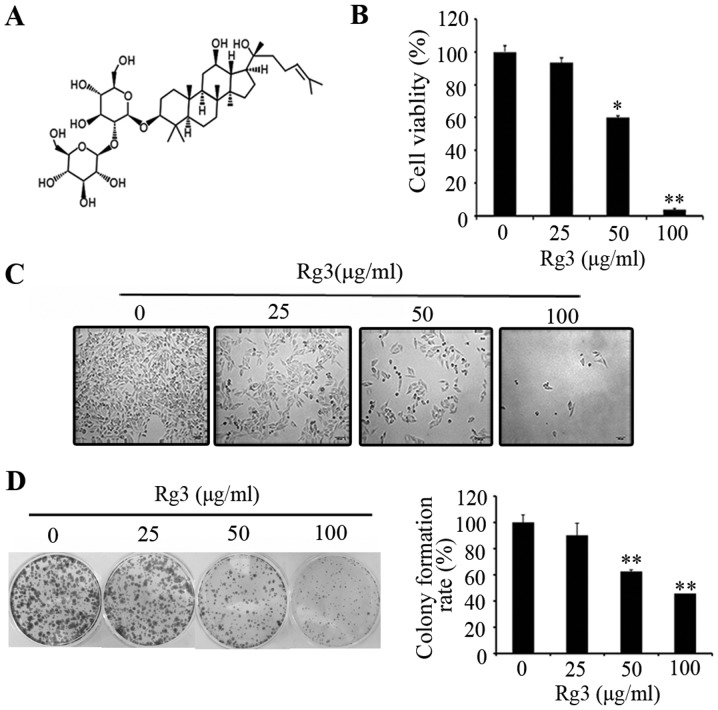
Rg3 inhibits cell growth in A375 melanoma cells. (A) Structure of 20 (R)-Ginsenoside Rg3. (B) A375 cells were treated with Rg3 (0, 25, 50 and 100 μg/ml) for 24 h. Cell viability was determined by MTT as described in Materials and methods. (C) Representative images and (D) long-term colony formation assay of A375 cells after treatment with Rg3 (0, 25, 50 and 100 μg/ml). Cells were grown in the absence or presence of Rg3 at the indicated concentrations for 7 days. Cells were fixed and stained with crystal violet. The results are representative of mean values from three separate experiments in triplicate. ^*^P<0.05; ^**^P<0.01.

In order to study the antitumor effect of Rg3 (structure is shown in Fig. 1A) on cell proliferation, melanoma cells were treated with Rg3 at different concentrations (0–100 μg/ml) for 24 h. We observed that Rg3 significantly inhibited cell proliferation in a dose-dependent manner as determined by MTT assay (Fig. 1B), representative images (Fig. 1C) of colony forming assay (Fig. 1D) are shown. There was a significant inhibitory effect of Rg3 on cell proliferation as compared with the untreated cells. These results demonstrate that Rg3 inhibits human melanoma cell proliferation.

### Rg3 decreases the expression of FUT4 and LeY

**Figure 2:**
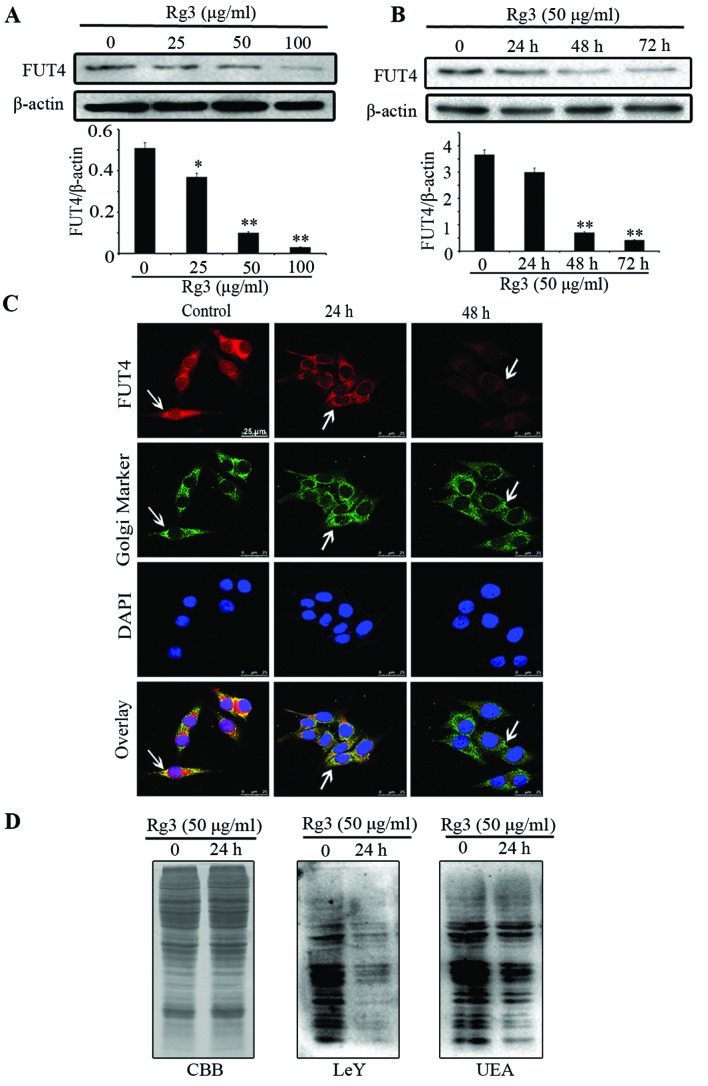
Rg3 decreases the expression of FUT4 and LeY. (A) Cells were treated with Rg3 (0, 25, 50 and 100 μg/ml) for 24 h or (B) treated with Rg3 (50 μg/ml) at different time intervals (0, 24, 48 or 72 h). Western blotting was employed to examine FUT4 protein expression. β-actin was used as an internal control, band density of three different western blots was analyzed (^*^P<0.05; ^**^P<0.01). (C) Cells were treated with Rg3 (50 μg/ml) for 0, 24 or 48 h. Immunofluorescence staining was used to detect the expression and cellular localization of FUT4 with a Golgi marker (Giantin antibody). Golgi region is labeled green, FUT4 is red. Co-staining of Golgi region and FUT4 were indicated with arrows in overlay (yellow). Bar, 25 μm. DAPI was used for counter stain. (D) Cells were treated with Rg3 (0 and 50 μg/ml) for 24 h. Western blotting was employed to examine the expression levels of LeY and UEA. Coomassie brilliant blue (CBB) staining of gels shows the comparable amounts of protein in each lane. The results are representative of three separate experiments in triplicate.

By western blotting (Fig. 2A and B), we found that Rg3 significantly inhibited FUT4 expression in a dose- and time-dependent manner. Moreover, confocal staining further confirmed the above results and showed that FUT4 was predominantly co-localized at position of Golgi apparatus with Giantin (Fig. 2C). To detect the protein expression of LeY and UEA lectin in cells treated without or with Rg3, Coomassie brilliant blue (CBB) staining and western blotting were employed (Fig. 2D). Western blotting revealed that Rg3 significantly inhibited the protein expression level of LeY and UEA lectin as compared with untreated cells. These results indicate that Rg3 inhibits the expression of FUT4 in a dose- and time-dependent manner. Furthermore, Rg3 effectively interferes with the synthesis of LeY antigen.

### Downregulating FUT4 expression inhibits A375 cell proliferation and decreases the expression of LeY

**Figure 3:**
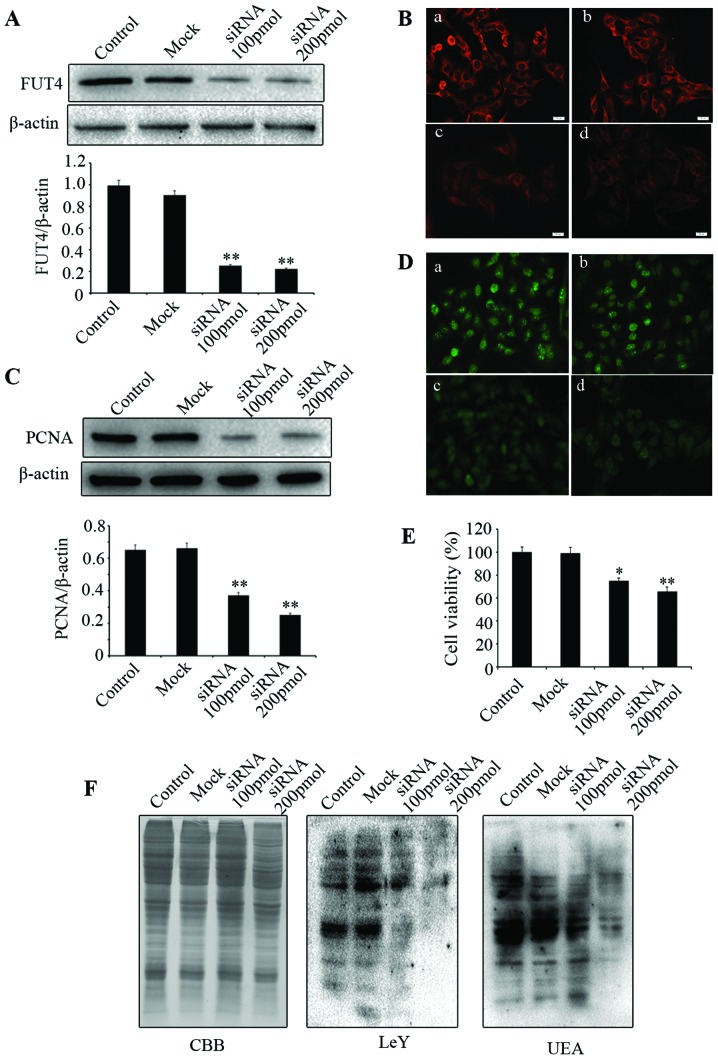
Downregulating FUT4 expression inhibits A375 cell proliferation and decreases the expression of LeY. FUT4 was detected by western blotting (A) and immunofluorescent staining (B) after transfection with FUT4 siRNA in A375 cells. PCNA protein expression was examined by western blotting (C) and immunofluorescent staining (D) after transfection with FUT4 siRNA in A375 cells. (a) Untransfected cells; (b) cells transfected with vector; and cells transfected with FUT4 siRNA (100 pmol) (c) or (200 pmol) (d). (E) Cell viability was determined by MTT. (F) Cells were transfected with FUT4 siRNA for 48 h. LeY and UEA expression was detected by western blotting. Coomassie brilliant blue (CBB) staining of gels shows the comparable amounts of protein in each lane. β-actin was used as an internal control, the statistical analyses of bend density for western blotting results are shown. The results are representative of three separate experiments. ^*^P<0.05; ^**^P<0.01.

To investigate whether downregulating FUT4 expression affects cell proliferation, western blotting and immunofluorescent staining were employed. PCNA is a commonly used cell proliferation marker. We showed that knocking down FUT4 expression with FUT4 siRNA (Fig. 3A and B) led to significant reduction of PCNA by western blotting (Fig. 3C) and immunofluorescence (Fig. 3D). MTT assay indicated that cell viability in FUT4 knockdown cells was significantly decreased in comparison with the mock treated and untransfected cells (Fig. 3E). Moreover, FUT4 siRNA transfection showed significantly decreased expression of LeY and UEA lectin as compared to mock treated and untreated cells (Fig. 3F). These results indicate that knockdown of FUT4 inhibits melanoma cell proliferation, which correlates to its inhibitory effect on the synthesis of LeY.

### Downregulating FUT4 expression decreases the tyrosine phosphorylation of EGF-mediated EGFR/MAPK

**Figure 4:**
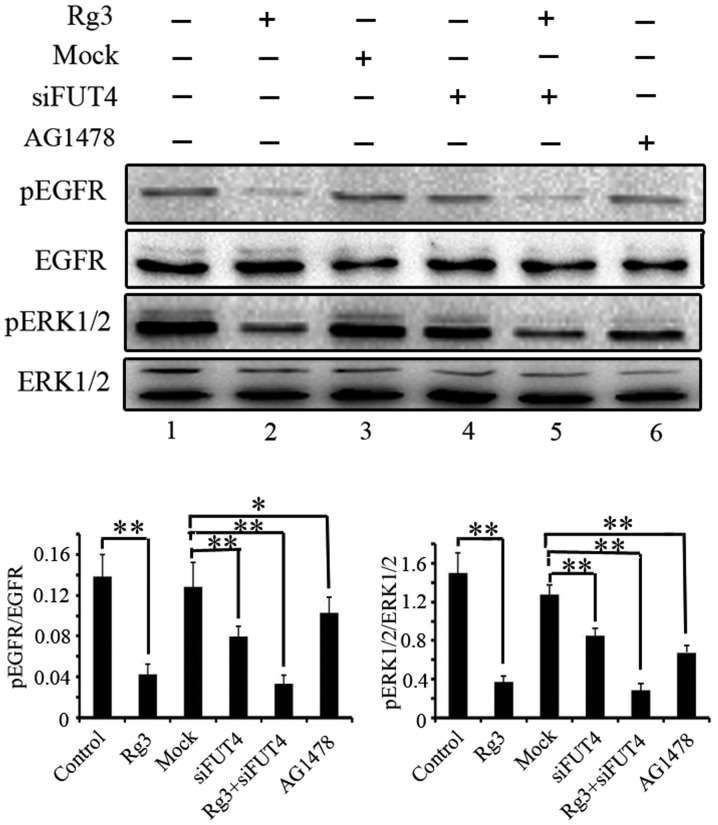
Downregulating FUT4 expression decreases the tyrosine phosphorylation of EGF-mediated EGFR/MAPK. A375 cells were treated with Rg3 (50 μg/ml), FUT4 siRNA (200 pmol), EGFR inhibitor (AG1478, 10^−4^ M) or FUT4 siRNA transfection followed by Rg3 treatment for 48 h. Control, untransfected cells; mock, cells transfected with vector control. Western blot analysis of expression levels of pERK1/2, ERK1/2, pEGFR and EGFR. The statistical analyses of bend density for western blotting results are shown. The results are representative of three separate experiments in triplicate. ^*^P<0.05; ^**^P<0.01.

The phosphorylation of epidermal growth factor receptor (EGFR) and extracellular signal-regulated kinases (ERK1/2) were examined to determine the inhibitory role of Rg3 on EGFR/MAPK pathway by western blotting. Cells were treated with Rg3, FUT4 siRNA and EGFR inhibitor (AG1478, 10^−4^ M) for 24 h. Pretreated with FUT4 siRNA and followed by Rg3 treatment for 24 h. Antibodies directed against pEGFR, EGFR, pERK1/2 and ERK, were used in western blotting (Fig. 4). Treatment with Rg3, FUT4 siRNA or Rg3 in combination with FUT4 siRNA significantly inhibited the phosphorylation of EGFR and ERK1/2 (Fig. 4, lanes 2, 4 and 5 vs. lane 1). Moreover, the EGFR inhibitor (AG1478) was also used to detect the EGFR/MAPK activation as an inhibitor control (Fig. 4, lane 6). The results indicate that the inhibition of the EGFR/MAPK pathway is at least part of the mechanism by which Rg3 inhibits melanoma cell proliferation.

### Rg3 inhibits the growth of melanoma xenograft tumors in vivo

**Figure 5:**
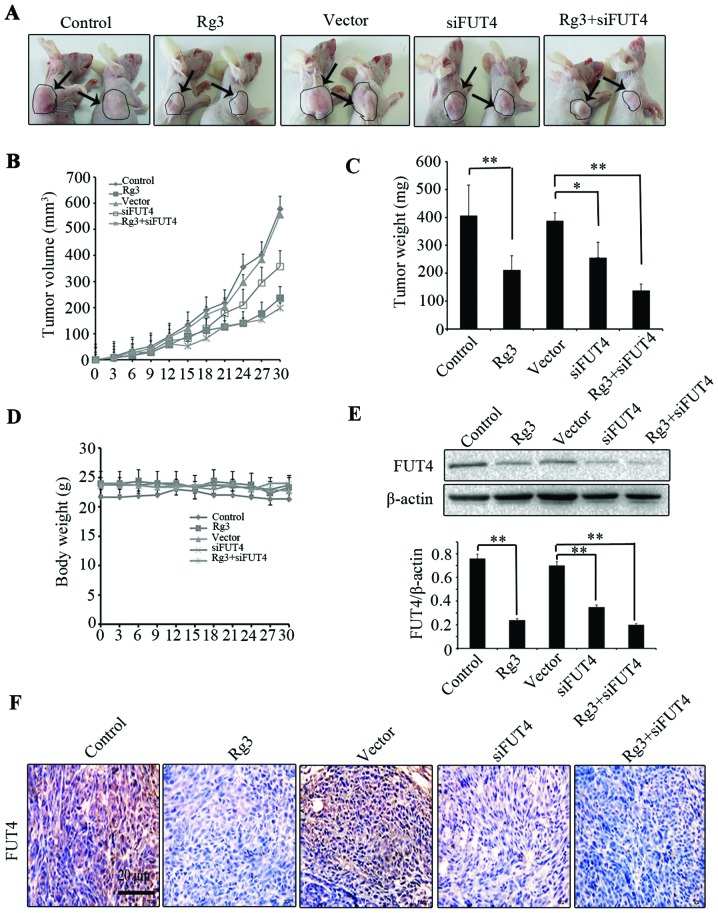
Rg3 inhibits the growth of melanoma xenograft tumors *in vivo*. The A375 cell xenograft nude mice were injected with vehicle or vector (control), Rg3 (20 mg/kg), siFUT4 (6 mg/kg) or Rg3 (20 mg/kg) combined with siFUT4 (6 mg/kg). (A) Sacrificed nude mice with xenograft tumor on day 30. Arrows indicate the location of the tumors; tumor volumes (B), tumor weight (C) and body weight (D) were generated with data from 6 mice in different groups. (E) Western blot analysis of expression levels of FUT4 in the tumor tissues from differently treated mice. β-actin was used as an internal control, the statistical analyses of bend density for western blotting are shown (^*^P<0.05; ^**^P<0.01). (F) Immunohistochemical staining of FUT4 expression in xenograft tumor tissues. Bar, 20 μm.

The antitumor effect of Rg3 on tumor growth was further validated *in vivo*. Mice were randomly divided into five groups for different treatments with six mice per group. Each treatment group was analyzed clinically (tumor volume, tumor weight and body weight) and each treatment on FUT4 expression was determined by western blotting and immunohistochemical staining. We found that Rg3 treatment group showed a significant inhibition of xenograft tumor volume by 52.50% (P<0.05), and the siFUT4 treatment group inhibited tumor volume by 35.79% (P<0.05); while Rg3 combined with siFUT4 treatment group inhibited tumor volume by 64.38% (P<0.01), indicating that greater inhibition was achieved than Rg3 or siFUT4 treatment group alone (Fig. 5A and B). The mice were sacrificed and tumor weight of each group on day 30 was compared. Rg3 (0.212±0.054 g) and siFUT4 (0.256±0.043 g) treatment groups showed low tumor weight as compared to vector and non-treated controls (0.406±0.110 g) (P<0.05). Moreover, combination treatment with Rg3 and siFUT4 led to much lower tumor weight (0.138±0.052 g) as compared to the mice treated with Rg3 or siFUT4 alone (Fig. 5C). There were no significant differences in body weight between control and treatment group (Fig. 5D). To investigate the mechanism of Rg3 inhibition on melanoma tumor growth, we analyzed FUT4 expression in Rg3, FUT4 siRNA or combination treatment group by western blotting and immunohistochemical staining. We found that Rg3 and siFUT4 treated group showed decreased FUT4 expression as compared to non-treated and vector-treated control group, while combinational treatment showed much lower expression of FUT4 compared with the mice treatment with Rg3 or siFUT4 alone (Fig. 5E and F). The results suggest that Rg3 inhibit melanoma growth *in vivo*.

## Discussion

Ginsenoside Rg3 is a monomer extracted from ginseng roots and it has strong antitumor activity. Previous studies have shown that Rg3 inhibits proliferation and induces apoptosis in gastric, hepatic and colorectal cancers (23,26,29,30), suppresses migration, invasion in lung cancer (28), enhances the susceptibility to docetaxel in colon cancer (31) and inhibits autophagy and sensitizes to doxorubicin in hepatocellular carcinoma (29). In the present study, we investigated the anticancer activity of Rg3 on human melanoma cells both *in vitro* and *in vivo*. We found that Rg3 inhibited melanoma cell proliferation in a dose-dependent manner. Moreover, Rg3 significantly reduced xenograft melanoma volume and weight when compared to the control group. These results indicate that Rg3 is a potential drug to inhibit melanoma proliferation.

The antitumor effects of Rg3 have been reported in many cancers, but whether Rg3 antitumor effect correlates to its regulatory effect on fucosylation is unclear. Several reports have shown that fucosyltransferases (FUTs) play a critical role on tumor progression. Yang *et al* proved that overexpression of FUT4 promoted A431 cell proliferation and inhibited cell apoptosis (32,33). Zhang *et al* found that suppression of FUT1/FUT4 expression by siRNA inhibited tumor growth (9). Ciolczyk-Wierzbicka *et al* demonstrated that higher expression of fucosyltransferases (FUT1, FUT4) played an important role in the formation of surface structures that facilitate metastasis of melanoma (34). In our study, we found that Rg3 inhibited melanoma cell proliferation through downregulation of FUT4 both *in vitro* and *in vivo*. Rg3 decreased the expression of FUT4 in a dose- and time-dependent manner. Moreover, Rg3 combined with FUT4 siRNA showed stronger effect than the treatment with Rg3 or FUT4 siRNA alone in melanoma xenograft models. These results suggest that Rg3 mediates inhibition of FUT4 expression and is involved in its inhibitory effect on cell proliferation. Rg3 is a potential FUT4 inhibitor and Rg3 combined with FUT4 siRNA may be a new therapy strategy in the treatment of melanoma.

The synthesis of LeY can be regulated at the transcriptional level by FUT4 (35). Abnormal elevation of LeY expression can be seen in many cancers correlating to malignant transformation. High expression of LeY promoted cell proliferation in ovarian cancer (8), decreased survival in lymph node negative breast carcinomas (36) and was also a strong risk factor for chemotherapeutic drug resistance in ovarian carcinoma patients (13,37). However, the antitumor effect of Rg3 on melanoma and its mechanism on FUT4 and LeY expression remains unknown. In this study, we demonstrated that Rg3 decreased the expression of FUT4/LeY and inhibited cell proliferation. Similar results were also achieved by knocking down FUT4 with its siRNA. Moreover, Rg3 combined with FUT4 siRNA showed greater inhibitory effect than the treatment with Rg3 alone. These results indicate that Rg3 inhibits FUT4 expression and FUT4 further reduces LeY synthesis, by which to inhibit cell proliferation through EGFR/MAPK signaling pathway. To our knowledge, we report for the first time the inhibitory role of Rg3 on human melanoma growth by reducing fucosylation.

EGFR can be directly or indirectly activated by different growth factors, which promote aberrant EGFR signaling activation and facilitate cell proliferation. Because of its role on tumor progression, the EGFR has been intensely studied as a therapeutic target (15). LeY is one of the glycoproteins carried by EGFR, changes of EGFR glycosylation may activate growth-factor receptor tyrosine kinases and promote tumor proliferation. LeY antibody (IGN311) inhibited EGFR-mediated MAPK signaling activation and prevented tumor growth (38). We have previously reported that FUT4 siRNA mediated deactivation of EGFR/MAPK pathway to inhibit cancer cell proliferation (9). Herein, we investigated the role of Rg3 regulated FUT4/LeY expression on EGFR-mediated MAPK signaling pathway. We found that Rg3, FUT4 siRNA and Rg3 combined with FUT4 siRNA led to reduced activation of EGFR and ERK1/2 in A375 melanoma cells.

In conclusion, our results suggest that Rg3 inhibits cell proliferation by downregulating the EGFR/MAPK signaling pathway through reducing FUT4/LeY expression. To our knowledge, this is the first study to evaluate the molecular mechanism of Rg3 on melanoma proliferation and the role of Rg3 regulated fucosylation changes on melanoma growth. Our results indicate that Rg3 may be a promising therapeutic drug for melanoma treatment.

## Abbreviations

FUT4: fucosyltransferase IV
LeY: Lewis Y
EGFR: epidermal growth factor receptor
MAPK: mitogen-activated protein kinases
ERK: extracellular regulated protein kinases
MTT: 3-(4,5-dimethylthiazol-2-yl)-2,5-diphenyltetrazolium bromide
TACA: tumor-associated carbohydrate antigen
